# DIGGER: exploring the functional role of alternative splicing in protein interactions

**DOI:** 10.1093/nar/gkaa768

**Published:** 2020-09-25

**Authors:** Zakaria Louadi, Kevin Yuan, Alexander Gress, Olga Tsoy, Olga V Kalinina, Jan Baumbach, Tim Kacprowski, Markus List

**Affiliations:** Chair of Experimental Bioinformatics, TUM School of Life Sciences Weihenstephan, Technical University of Munich, 85354 Freising, Germany; Chair of Experimental Bioinformatics, TUM School of Life Sciences Weihenstephan, Technical University of Munich, 85354 Freising, Germany; Helmholtz Institute for Pharmaceutical Research Saarland (HIPS), Helmholtz Centre for Infection Research (HZI), 66123 Saarbrücken, Germany; Chair of Experimental Bioinformatics, TUM School of Life Sciences Weihenstephan, Technical University of Munich, 85354 Freising, Germany; Helmholtz Institute for Pharmaceutical Research Saarland (HIPS), Helmholtz Centre for Infection Research (HZI), 66123 Saarbrücken, Germany; Faculty of Medicine, Saarland University, 66421 Homburg, Germany; Chair of Experimental Bioinformatics, TUM School of Life Sciences Weihenstephan, Technical University of Munich, 85354 Freising, Germany; Department of Mathematics and Computer Science, University of Southern Denmark, 5230 Odense M, Denmark; Chair of Experimental Bioinformatics, TUM School of Life Sciences Weihenstephan, Technical University of Munich, 85354 Freising, Germany; Chair of Experimental Bioinformatics, TUM School of Life Sciences Weihenstephan, Technical University of Munich, 85354 Freising, Germany

## Abstract

Alternative splicing plays a major role in regulating the functional repertoire of the proteome. However, isoform-specific effects to protein-protein interactions (PPIs) are usually overlooked, making it impossible to judge the functional role of individual exons on a systems biology level. We overcome this barrier by integrating protein-protein interactions, domain-domain interactions and residue-level interactions information to lift exon expression analysis to a network level. Our user-friendly database DIGGER is available at https://exbio.wzw.tum.de/digger and allows users to seamlessly switch between isoform and exon-centric views of the interactome and to extract sub-networks of relevant isoforms, making it an essential resource for studying mechanistic consequences of alternative splicing.

## INTRODUCTION

Alternative splicing (AS) refers to differences in the processing of transcripts (e.g. exon skipping, intron retention etc.) allowing to synthesize different protein variants from the same gene. These protein variants, called isoforms, can vary in their functionality or even have opposite roles (1). This mechanism is important in cell development and differentiation (2) but also in diseases such as cancer (3), heart and kidney diseases (4,5).

Protein-protein interaction (PPI) networks such as BioGrid (6) or STRING (7) are an important resource in systems biology. PPI interactions are identified in tedious experiments, mostly via affinity purification mass spectrometry or yeast two hybrid screens (8). Due to the high number of possible interactions (quadratic in the number of considered proteins), efforts are limited to testing only major protein isoforms, hence neglecting the considerable influence of AS on the interactome. For instance, it was shown that AS remodels the network of PPIs in a tissue-specific manner (9) and that protein variants from the same gene differ in their interactions due to changes in the structural domain composition (1,10). Yang *et al.* found that most isoforms share <50% of interactions and only 21% of isoforms pairs have identical interaction profiles (1). Furthermore, a high proportion of these isoforms are known to be expressed in a tissue-specific manner (11). Recently, Climenté-Gonzalez *et al.* showed that around 30% of all isoform switches in tumor cells affect domains that mediate protein interaction (12). This suggests a widespread impact of AS in the human interactome that is currently neglected (13).

Domain-domain interaction (DDI) databases provide an annotation of PPIs in a structural context. This structurally resolved interactome is frequently used to analyze the location of disease mutations in proteins (14). 3did visualizes DDIs as a graph but does not integrate this information with experimentally validated PPIs (15). In contrast, Interactome3D and INstruct add structural details such as DDIs and residues to the PPI networks but do not project this information to the level of isoforms or exons (16–17). Given the resolved structural composition of different isoforms, this annotation can be extended to predict isoform-specific interactions consistent with experimental results (1,18). It is further possible to identify residues located at the interface of a PPI to study PPI perturbation (19). However, existing efforts are mostly focused on studying mutations that perturb these interactions (20–21) but do not consider consequences of AS. Few existing tools address this gap to systematically study AS. The Cytoscape app DomainGraph (22) visualizes domain interactions simultaneously with protein interactions and analyzes the effect of differential exon usage. However, DomainGraph is limited to the output of the tool AltAnalyze (22). Ghadie *et al.* developed DIIP using a similar method to predict an isoform interactome (18). While their results were verified based on the experimentally validated isoform interactome reported by Yang *et al.* (1), their database covers only a fraction of the proteome with 2944 reference proteins and 4363 interactions. Exon Ontology (EXONT) characterizes protein domains and features that are affected by AS (23) but does not consider AS on the network level.

PPIXpress extends this idea to construct a condition-specific PPI network based on transcript expression (24). While covering the entire proteome, it was not intended for studying individual genes or protein variants. Neither DIIP nor PPIXpress provide a graphical visualization or support the analysis of a single splicing event such as the gain or loss of a domain. Furthermore, existing tools do not allow a side-by-side comparison of interactions of different protein isoforms, which, however, is crucial to understand the functional effect of an isoform switch between two conditions. To close this gap, we developed DIGGER (Domain Interaction Graph Guided ExploreR), a user-friendly database and web tool to explore the functional impact of AS on human PPIs. In contrast to existing tools (Supplementary Table S1), DIGGER includes residue-specific information, highlights consequences of exon skipping events, visualizes interactions between multiple isoforms and offers a user-friendly web interface.

## MATERIALS AND METHOD

### Joint PPI and DDI network

**Figure 1.**
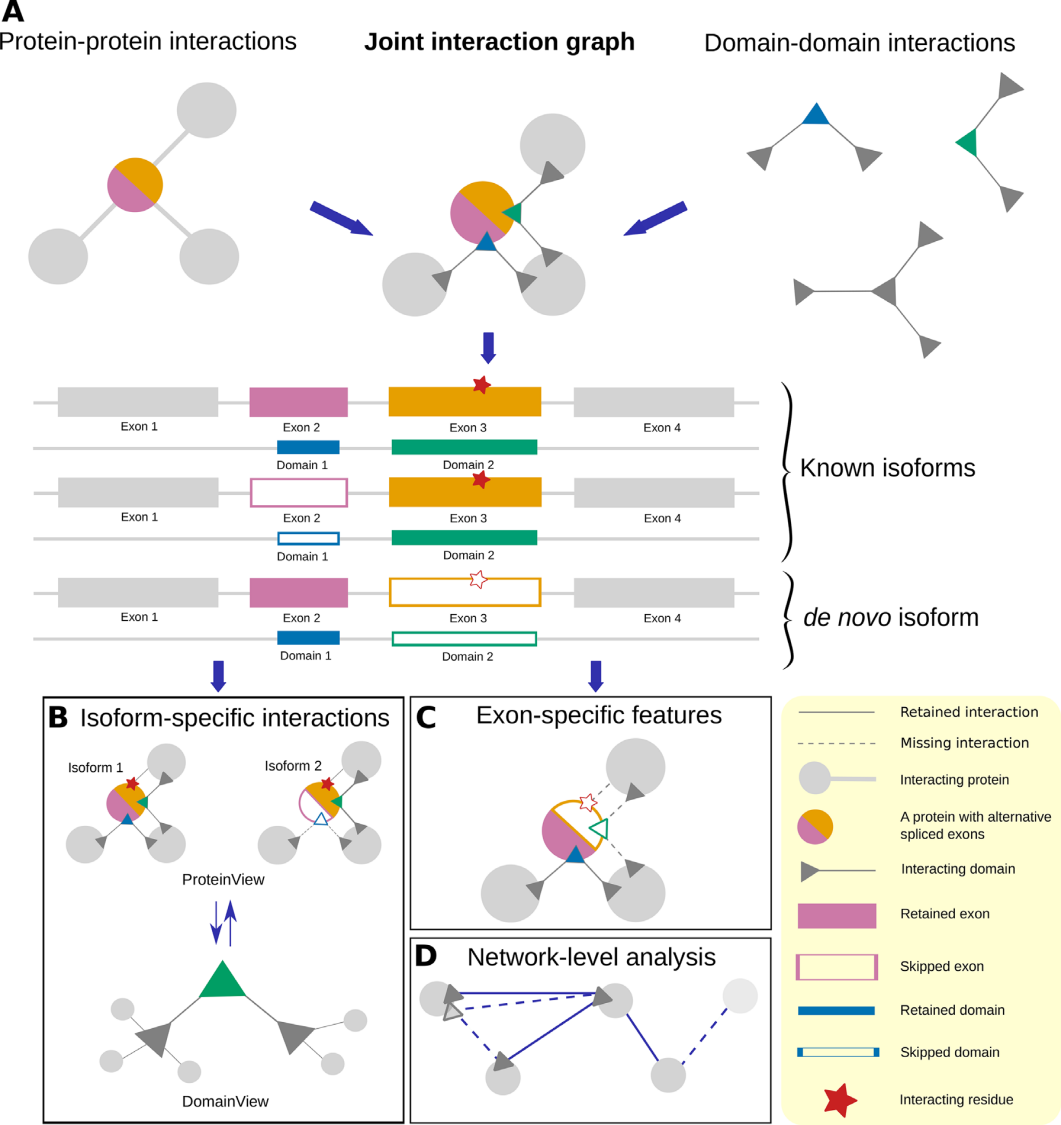
(**A**) Protein–protein interaction data is integrated with a domain–domain interaction data to construct structurally annotated interactions for every gene. This annotation is then used to compare between different protein variants in isoform-level analysis mode (**B**) and to identify the functional effect of a skipped exon in exon-level analysis mode (**C**). In the latter, residues located at the corresponding interaction interfaces are highlighted. Network-level analysis (**D**): DIGGER generates a subnetwork from a list of protein isoforms (see network-level analysis for details).

The human PPI network with 24 969 reference proteins and 410 961 interactions was obtained from BioGRID version Homo_sapiens-3.5 (6) and DDIs were downloaded from 3did (v2019_01) and DOMINE (v2.0) (15,25). 16,094 low-confidence interactions from DOMINE were removed. The remaining 2989 high- and 2537 mid-confidence interactions were integrated with all 13 499 reported interactions in 3did to obtain 17 349 interactions between 8190 Pfam domains (26). We implemented a joint network graph (Figure 1) that integrates PPIs and DDIs and in which nodes represent protein domains defined by concatenating Entrez and Pfam id. The edges between the nodes represent DDIs which are defined if the domains are known to interact and if the respective proteins are also PPI partners. The joint graph greatly speeds up the real-time processing of the requested data and can also be useful for studying the interacting regions of the proteins in other studies. Hence, we make the joint graph available as download in multiple formats on the DIGGER website.

### Position-specific PPI network construction

**Figure 2.**
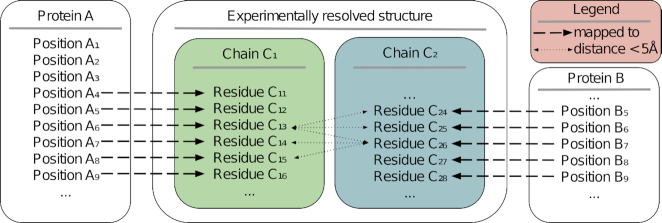
Schematic representation of the construction of the position-specific protein–protein interaction network. Identification of an interaction between proteins A and B based on their mappings to two different chains C1 and C2 in the same experimentally resolved structure. For example, the amino acid at position A_6_ of protein A is defined to interact with the amino acids at positions B_5_, B_6_, and B_7_ of protein B and vice versa.

We constructed a PPI network of the human proteome based on experimentally resolved structures in the Protein Data Bank (PDB) (27). First, we mapped individual amino acid positions to individual residues in experimentally resolved protein structures. To this end, we aligned the sequences of all protein isoforms in the human proteome to all protein chains with >95% sequence identity in the PDB. The second step was the identification of all interaction partners of a particular amino acid residue, which we defined as all amino acid residues from other protein chains co-resolved in the same three-dimensional structure and <5Å from the residue of interest (Figure 2). In total, we could identify 8991 DDIs, 3230 of which are also covered by BioGRID. Since a protein can be mapped to multiple structures, a single amino acid can be involved in multiple interactions with residues belonging to different interaction partner proteins. For proteins that have been experimentally resolved in complex with other human proteins, we can thus map every residue on the PPI interface to a particular position in the genome, and hence to a particular exon. Additionally, we obtain the same information for the interacting protein(s), creating a position-specific picture of the PPI interface.

### Mapping of protein domains to exons

The exons and the domain composition of annotated proteins were obtained from Ensembl 99 using the Biomart webtool (28). We generated database tables for both genomic data, e.g. genes with their corresponding transcript and exon coordinates, and for proteins, e.g. isoforms and their domains. We converted the protein coordinates to genomic coordinates in the coding sequence and merged both tables to be able to map transcripts with their corresponding exons to the corresponding protein isoforms and Pfam domains. We further constructed a database table that maps position-specific residue annotations to exons to obtain an exon-level PPI network. The Biomart mapping table was also used to convert between Entrez, Ensembl and Uniprot ids. All data tables are available as downloads on our website.

### RNA-Seq dataset analysis

The transcript expressions using RNAseq were obtained from the Cancer Genome Atlas pan-cancer dataset downloaded via the Xena Browser (29) (https://xenabrowser.net/datapages/) for the sample identifier TCGA-S9-A7J2-01. The isoform expression levels are originally estimated based on RSEM (30). In this analysis, all transcripts with an expression value above 1.0 are considered as abundant. The source code for the analysis is available at (https://github.com/louadi/RNA-Seq-DIGGER).

### Web interface

DIGGER was developed using the Python web framework Django and is released as open source under the GPLv3 license (https://github.com/louadi/DIGGER). For visualization, we used the Javascript library vis.js (https://visjs.org/) with different graph layout parameters depending on the size of the generated network.

## DATABASE CONTENT AND APPLICATIONS

DIGGER integrates the interactome from BioGRID (6) with DDIs of Pfam domains reported by DOMINE and 3did (15,25), comprising 9370 reference proteins and 52 083 PPIs that are confirmed by at least one DDI and 17 390 PPIs mediated by multiple DDIs. Notably, none of the existing resources annotate individual exons, which we consider a prerequisite to study the consequence of AS on DDIs. To mitigate this, DIGGER provides a unique mapping of interface residues of interacting proteins to exons based on experimentally resolved structures in the Protein Data Bank (PDB) (27). We generated a PPI network resolving interactions on a residue-specific level, i.e. for each protein residue on an interaction interface, we derived information on all residues from the interacting protein that is in contact with it (see Materials and Methods for details). In this way, genomic information on a splicing event can be directly mapped onto protein three-dimensional structure and the impact of the AS event on the PPI interface can be assessed. Through DIGGER’s user-friendly web interface, researchers can interactively visualize the domain composition for any protein isoform, with detailed information of the interacting domains between the selected protein and of its partners in the PPI network.

DIGGER offers three different modes (Figure 1) that can be used interchangeably. Here, we explain these modes individually and provide several use cases.

### Isoform-level analysis

In this mode, users can query a protein isoform and visualize its composition including the exons and their corresponding domains as well as residues predicted to be part of the interface to interacting proteins. Interactions specific to the selected isoform are displayed as an interactive graph where users can toggle between the ProteinView and the DomainView to visualize interacting proteins or domains, respectively. Importantly, the ProteinView will highlight missing domains, i.e. domains that are not annotated in Pfam for a given isoform.

Protein domains are often shared between different isoforms. The DomainView (Figure 1B) highlights domain-domain interactions together with potential protein interaction partners that utilize this domain and can be considered as a domain-specific interactome independent of the associated protein. This view is not only useful to study spliced domains but can also be extended for other applications such as studying coding disease variants affecting a protein domain or analysing specific drugs targeting a domain unit.

#### DIGGER scores multi-domain interactions to account for limited evidence

In contrast to existing methods that only consider a PPI missing if all its supporting DDIs are missing, DIGGER provides a score representing the percentage of missing domains for every interaction in a PPI. This allows for more fine-grained considerations and hence better control of the tradeoff between false positive and false negative PPIs. As an example for the usefulness of this feature, we consider a data set of 19 genes with 46 experimentally verified isoform-specific interactions (1). DIGGER could confirm 36 out of 46 experimentally verified splicing events that disrupt interacting domains (Supplementary Table S2). In 10 non-identified cases, no high quality structural annotated interactions were reported for the spliced domains. In one case, we observe that an isoform of CDK5 with a duplicated kinase domain interacts with the protein CCND2, while in another variant with only a single kinase domain, this interaction is missing.

**Figure 3.**
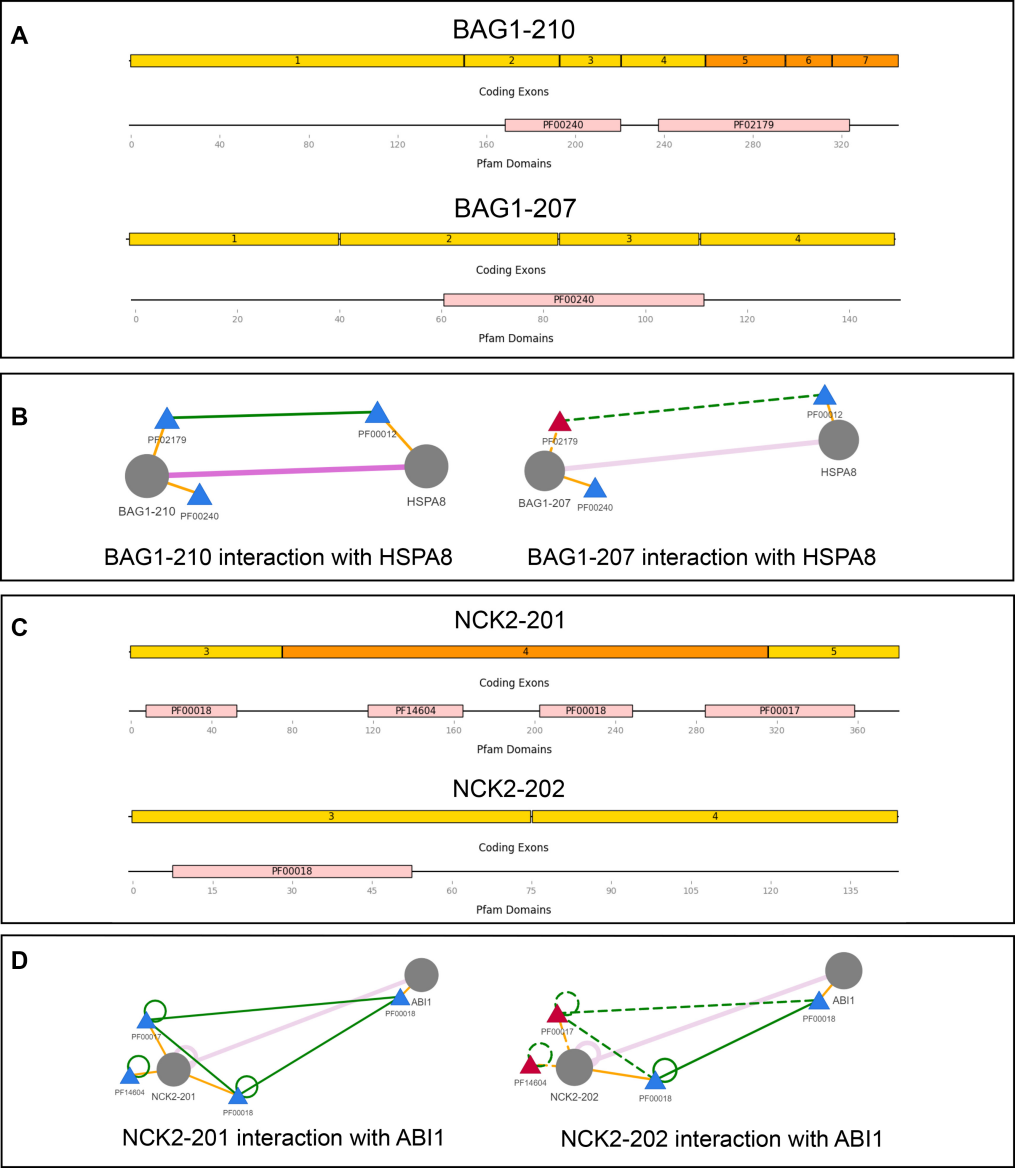
InteractionView example for comparing different isoforms. Circles represent proteins and triangles represent domains. A and B represent a comparison between the exon and domain structure of two isoforms of the gene BAG1 and their interaction with the protein HSPA8. (**A**) Isoform BAG1–207 lacks the domain PF002179 while for BAG1–210 both domains are preserved. (**B**) The effect of losing domain PF002179 is highlighted in the network by red triangle nodes and dashed edges. Notably, PF002179 is the only domain mediating the interaction with a domain of the protein HSPA8 suggesting that this interaction is missing for isoform BAG1–207. In the second example (**C**), two domains mediate the interaction between two proteins NCK2 and ABI2. (**D**) As one of them is spliced out for isoform NCK2–202, the interaction is scored 0.5 for this isoform and 1.0 for NCK2–201. Missing exons such as exon number 5–7 in BAG1–210 (**A**) are shown in orange if residues are predicted to be on the interface. In this case, the interface with HSPA8 is mapped to the exon 5 and also supported by residue-level evidence.

Figure 3 illustrates two examples from the subset where isoforms of the genes BAG1 and NCK2 are shown to lose PPIs with their partners due to alternative domain usage. The first example (Figure 3A and B) shows that the interaction between the proteins BAG1 and HSPA8 is mediated by only one of the two domains of BAG1 (the BAG domain PF02179). This interaction is also confirmed by residue-level information. In contrast, we observe that for the interaction between NCK2 and ABI1 (Figure 3C and D), two domains of NCK2 participate in the interaction (SH2 domain PF00017 and SH3 domain PF00018), but the loss of the SH2 domain interaction disrupts the PPI.

This observation highlights a limitation of the current practice where an interaction is only considered as missing if all domain-domain interactions are missing (18,24). The exact domain(s) that mediate a PPI can not be precisely identified when multiple domains interact between two proteins as in the above example where the loss of domain SH2 alone is sufficient to disrupt the interaction (1). In total, we found 25 isoform-specific interactions that are mediated by multiple domains reported in (1), which motivates DIGGER’s approach of scoring interactions rather than filtering them following an all or nothing strategy. The scores are available in a downloadable table in InteractionView.

### Exon-level analysis

We propose that an exon-level view on PPIs and DDIs is best suited to recapitulate the effect of AS on the interactome. Thus, DIGGER maps domains and interface residues for all protein variants of a single gene to genomic coordinates and corresponding exons (Figure 1A–C, see Materials and Methods for details). In contrast to isoform-level analysis, the exon-level analysis mode allows the user to identify any domains encoded by the exon of interest and to visualize the interaction mediated by them. This method allows investigating the consequences of a putative or observed exon loss. For a better comparison, we also linked this feature with the isoform-level analysis by listing protein variants that contain the exon of interest. The user can, in a similar way to the previous modes, visualize all interactions of every partner individually, where the percentage of missing putative interactions is shown as a percentage score. Here, a missing domain is defined as any domain with a sequence overlap with the selected exon. To compare different possible scenarios resulting from different isoforms, the user can also visualize every DDI individually using the DomainView (Figure 1B).

**Figure 4.**
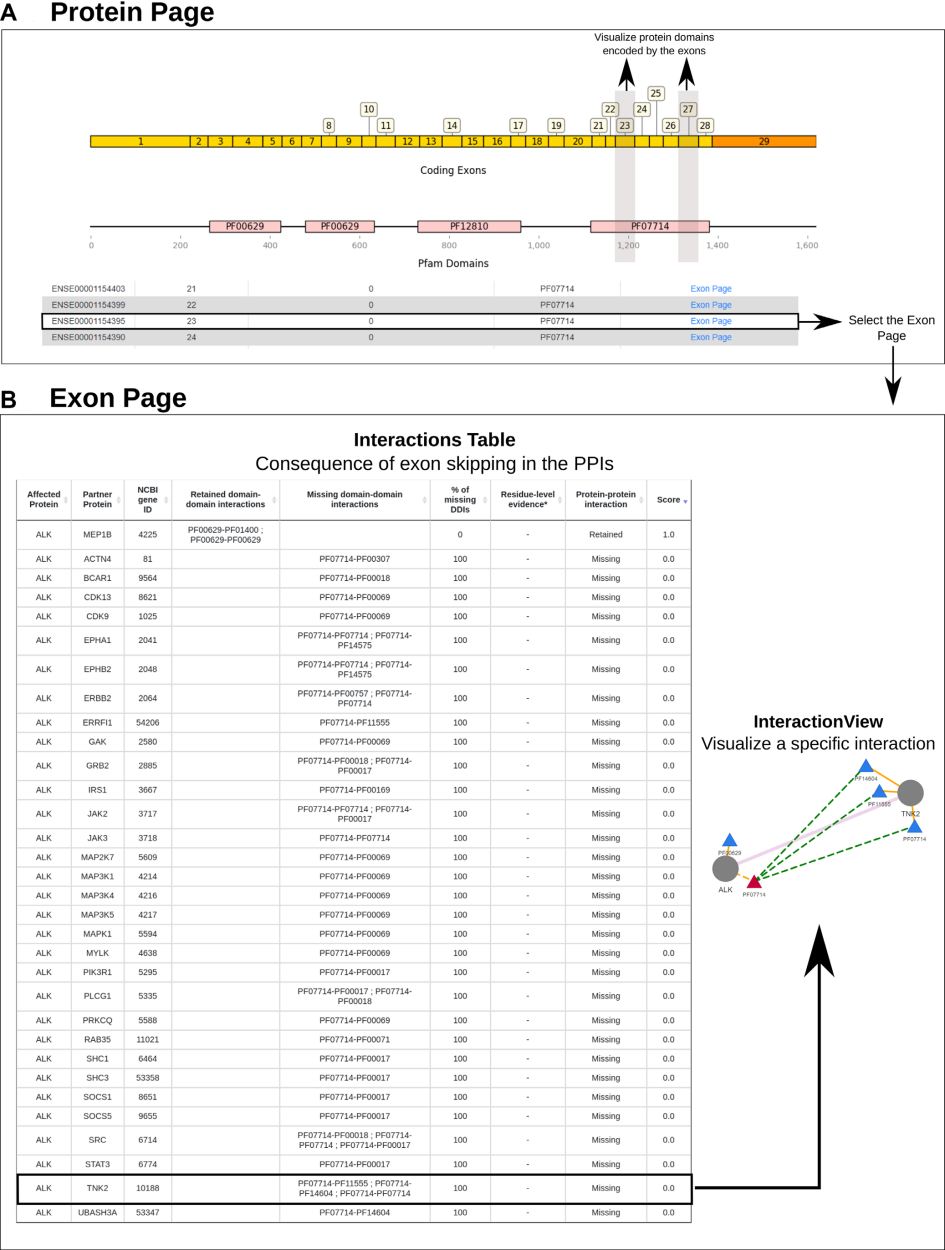
DIGGER can be used to study the putative effect of an exon skipping event. (**A**) in this use case, we consider an event resulting in a non-annotated protein. (**B**) We continue with exon-level analysis to show affected domains and interactions of the resulting protein. The dashed edges represent the interactions of a spliced domain that is encoded by the selected exon.

DIGGER is unique in that it goes beyond domain-level annotation of PPIs to exon-level structural evidence of an interaction. An exon is considered to have structural evidence for a PPI if it codes for residues that are found within a distance of <5 Å in a co-resolved structure of the two proteins (see Materials and Methods for details). To run this mode, the user can input an exon Ensembl ID or a gene ID followed by the coordinates of the exon in hg38, which is similar to the output produced by most AS event detection tools. Another option to access this mode is from isoform-level analysis mode, by selecting a protein and then choosing a specific exon from the exon or domain structure view (Figure 4).

#### Use Case 1: Truncated isoforms of anaplastic lymphoma kinase lose 97% of their PPIs due to AS

We demonstrate how explorative analysis in DIGGER can be used to create hypotheses or to interpret experimental results with respect to molecular consequences of alternative splicing. As a case study, we consider experimentally verified splicing variants of the tyrosine kinase receptor family.

In non-small cell lung carcinoma, Lobo de Figueiredo-Pontes *et al.* reported non-functional isoforms of anaplastic lymphoma kinase (ALK) that lack a functional kinase domain due to skipping of exons 23 and 27 (31). Figueiredo-Pontes *et al.* found that these isoforms are still able to fuse with EML4 but because of the lack of the kinase domain, the dimer EML4-ALK was unable to phosphorylate tyrosine sites. We can assess the consequence of skipping these exons using DIGGER’s visualization, where we observe that no annotated isoform lacks exon 23 or 27 in our database or in the Ensembl transcript database. In ALK-201, the main isoform of the ALK gene, exons 21 to 28 encode for the domain tyrosine kinase (PF07714 in Figure 4A). By choosing the exon page (see exon-level analysis) of any of the two exons, we can contemplate the effect of losing one of these exons on ALK PPIs. Strikingly, the deletion of either exon 23 or 27 affects 31 of the 33 known structurally annotated interactions of the ALK gene (Figure 4B). This corroborates the experimental results showing that skipping of these exons leads to a translated but non-functional variant which likely lost 97% of its PPIs.

In another interesting example, Ellis *et al.* (9) found that the ability of gene GRB2 to self-interact was lost by deletion of a tissue-specific exon that overlaps with SH2 domain (PF00017) while the interaction with RAPGEF1 was retained. DIGGER could confirm that the self-interaction is mediated by the SH2 domain while the interaction with RAPGEF1 is mediated by SH3 domain (PF00018) and thus not affected in the isoform missing this exon.

#### Use case 2: AS leads to different insulin response

**Figure 5.**
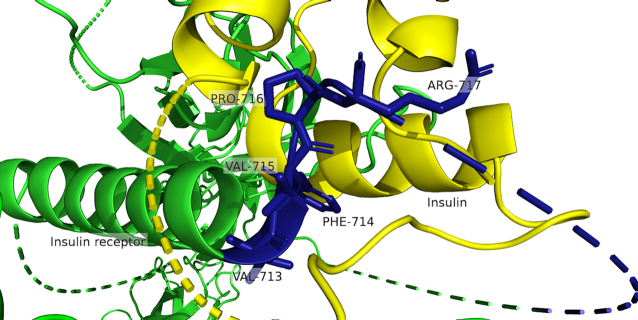
Three-dimensional structure of a complex with insulin (yellow) and the insulin receptor (green) (PDB id 6PXV, not resolved parts are drawn as dotted lines). The residues that are part of exon ENSE00001157509 (blue) are forming an interaction interface between the insulin receptor and insulin. Although only five residues (chain A 713–717) are resolved in the structure, they prove that the interface can be affected by the deletion of the exon ENSE00001157509.

Denley *et al.* investigated two isoforms of the insulin receptor gene that respond differently to insulin (32,33), namely INSR-201 (ENST00000302850) and INSR-202 (ENST00000341500), which differ by the absence of exon 11 from the isoform INSR-202. Since the amino acids encoded in the skipped exon 11 (ENSE00001157509) are not a part of any annotated domain, we explored the existence of any known protein motifs. We found that the exon encodes the PKA phosphorylation site (MOD_PKA_1) according to the Eukaryotic Linear Motif resource (34), suggesting that a post-translational modification may be affected with possible consequences for protein signalling. Interestingly, DIGGER’s exon-level analysis shows that this exon also contains residues that interact with four insulin isoforms (Figure 5). Consequently, the exon-level analysis results suggest that this interaction will be affected by skipping this exon. This observation shows the importance of residue-level interaction data as a complement to domain interactions and confirms the utility of this new feature that could be used to generate hypotheses of possible scenarios resulting from exon skipping.

### Network-level analysis

To study the effect of AS on PPIs and DDIs on a larger scale in systems and network biology, it is crucial to consider interactions between multiple protein isoforms or domains in a comprehensive view. Typical examples are the in-depth analysis of AS-driven interaction changes in a protein complex or a list of differentially expressed (or spliced) genes or proteins from transcriptomic or proteomic experiments. PPIXpress (24), the only other tool that constructs a subnetwork based on a list of transcripts, does not offer visualization of the network, affected edges or interacting domains. In contrast, DIGGER visualizes interactions between multiple proteins or isoforms. Users can input a list of gene, transcript or protein Ensembl identifiers to construct a subnetwork.

**Figure 6.**
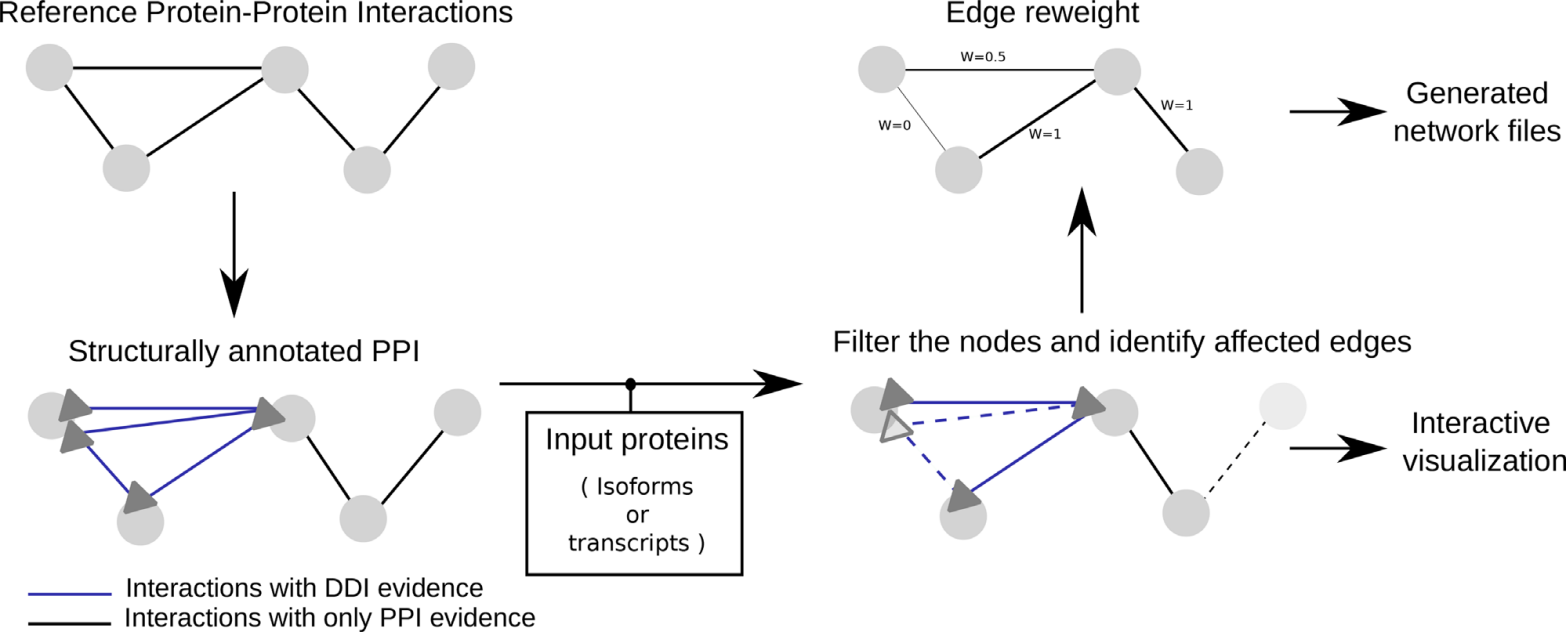
In network-analysis mode, DIGGER highlights domains absent in the user-submitted isoforms and identifies missing interactions mediated by these domains. Edges are scored according to the ratio of missing putative interactions. Results can be visualized or exported for further analysis in third party tools.

As illustrated in Figure 6, DIGGER generates a subnetwork of interactions showing domains putatively mediating these interactions. The interaction is labeled ‘PPI’, if there is no structural evidence for it. Otherwise, it is labeled ‘PPI-DDI’ and the specific DDIs are shown by one or multiple edges. Analogous to the isoform-level analysis, we provide a score for each interaction based on the fraction of annotated DDIs that are present. When the resulting network is exported, the score provides an edge weight for subsequent analysis.

Applying network-level analysis to the RNA-seq data from The Cancer Genome Atlas pan-cancer dataset (35), we could identify 41 449 edges with one or more DDIs, of which 3258 show at least one missing domain and 2,088 putative interactions that are likely completely missing. The details and code for this analysis can be found in the Materials and Methods section. These results corroborate the need for transcript and isoform-level network analysis to better reflect the proteome in disease-relevant conditions such as cancer.

## DISCUSSION

With DIGGER, we present a versatile, user-friendly database and web tool to study the impact of AS on PPIs. DIGGER integrates PPI and DDI interactions into a joint graph and, as a key innovation, maps interacting residues to exons, allowing us to better assess the functional consequence of AS. Our analysis based on isoform-, domain- and exon-specific views of the human interactome shows a widespread effect of AS in concordance with experimental data. DIGGER is the first tool to score isoform-specific interactions based on the ratio of missing DDIs which facilitates the interpretation of interactions involving multiple domains. To facilitate systems and network biology analyses, DIGGER constructs a subnetwork of the joint PPI and DDI graph based on a list of isoforms or protein variants. Using this network-level analysis mode, it is possible to visualize affected DDIs. The resulting network can be exported for further analysis, e.g. for comparing different conditions. However, it is important to bear in mind that some interacting isoforms in the subnetwork are not necessarily co-expressed in the same condition or tissue. The user can use this mode with RNA-Seq data (Option 2 in DIGGER Network-level analysis) to extract expressed transcripts and explore the specific interactions between them.

In addition to the visualization, DIGGER offers the following benefits over PPIXpress via its network analysis functionality. First, in PPIXpress a PPI is only considered missing if all associated DDIs are missing. In contrast, DIGGER allows more flexibility by offering a filter option based on the ratio of missing interactions. Note that filtering all interactions with weight equal to 0 will be equivalent to the PPIXpress algorithm. Second, PPIXpress only considers the most highly expressed transcript, which is arguably an oversimplification. In contrast, DIGGER combines all structural information from different isoforms of the same gene. As a result, missing domains are defined as those missing in all protein variants in the input list but known to be present in other variants that were not included. Again, users can choose to include one transcript or isoform from each gene, e.g. the most highly expressed one to obtain comparable results to PPIXpress. We believe that these improvements provide the user with considerably more flexibility and better interpretability of the results for in-depth analyses on the system or network level.

The general workflow of DIGGER provides the user with an easy and interchangeable navigation between these modes and the different views (Supplementary Figure S1). As a result, DIGGER is the only database that allows for a complete exploration of AS impact from the exon to the network level. In contrast, comparable tools and methods cover only individual aspects, such as the Ghadie *et al.* method (18) and DomainGraph (22) that only focus on the isoform interactions or PPIXpress that analyzes transcript expression data (24). Furthermore, DIGGER is the only resource that combines DDIs with residue specific interactions to identify the consequence of skipping an exon.

We could show that DIGGER’s ability to map interacting residues to exons enables us to study splicing events that result in hitherto unannotated protein isoforms with experimental evidence. While this is a powerful approach to assess the potential impact of alternative splicing events between two conditions, we caution that the structures used for this annotation are typically derived from the full-length transcript and mostly limited to the major isoform. They thus do not reflect the influence of the exon itself on protein folding. Nevertheless, identifying putatively interacting residues as well as domains encoded by the exonic region allows for exploring all possible scenarios that result from AS events such as exon skipping.

Naturally, the annotations found in DIGGER are limited by the quality of the integrated PPI and DDI data sets as well as the quality of the structural annotations of domains and residue interfaces. Currently, DIGGER covers 37% of the proteins and 13% of the interactions in BioGRID. Although the majority of proteins are annotated with at least one domain (26), the experimental coverage of DDIs is comparably poor. Furthermore, the DDI view of interactions neglects interactions mediated by disordered regions. Moreover, AS events occurring in these exons can possibly alter the translation or the folding of the protein. The function role of these exons is still not very well understood and even controversial (36–37). In the current database, around half of the annotated exons map to disordered regions (48% of the 307 219 annotated exons from protein coding transcripts) which limits the efforts towards a complete structurally annotated isoform interactome. By incorporating residue-level evidence, we increased the structural coverage by 4968 exons that were initially mapped to a disordered region. Another way to approach this problem is to explore linear binding motifs that could further expand our understanding of the rule of individual exons in the PPI. Thus, we plan to incorporate protein binding motifs in a future release of DIGGER and to integrate further data sources for DDIs and PPIs such as STRING (7).

Another challenge in the field is to determine the exact domains or exons responsible for a PPI when multiple domains are mapped to the interaction interface. Our analysis shows that 17 390 PPIs are annotated with multiple DDIs (33% of the structurally annotated PPI). Identifying the AS impact on these interactions is more difficult, since the role of individual domains or exons is not clear. To mitigate this, DIGGER scores the percentage of isoform-specific interactions missing associated domains. Here, users should be careful when choosing a threshold to avoid an excess in false positives or false negatives. Additional experimental results on isoform-specific interactions are needed to resolve this and to determine the best possible threshold. Another possibility to narrow down the regions corresponding to the interacting surfaces between the two proteins is the use of residue-level evidence provided in DIGGER at the exon-level. The existence of an interacting residue in a single specific interface provides strong support that the interaction is specific to that domain (or exon).

## CONCLUSION

Recent studies emphasize the considerable influence of AS on human PPIs. As discussed previously by Talavera *et al.* (38), this may lead to a significant bias in network-driven systems biology analysis. For every PPI, there is a potentially large number of isoform combinations that would have to be experimentally validated (2). Given limited experimental data, it is essential to build computational approaches to distinguish between protein isoforms and to identify the function and interactions of putative new variants. DIGGER closes this gap in order to help biomedical researchers to address the complexity in visualizing and analyzing the functional impacts of AS in a user-friendly fashion and on multiple levels, ranging from protein isoforms, via domains, down to exons. DIGGER integrates state-of-the-art annotations of PPIs and DDIs and enriches them with a novel approach to gain residue-level information of PPI. We have shown that the results generated by DIGGER are consistent with experimental evidence in the context of isoform-specific interactions and exon skipping. DIGGER is ideally suited to investigate the differences between isoforms, to analyse the effect of an isoform-switch, or to explore how alternative splicing events such as exon skipping lead to altered interactions of protein isoforms. DIGGER provides a basis for network analysis, by re-weighting the reference PPI based on the structural evidence of the specific interacting proteins. In the future, we envision to extend DIGGER to provide network analysis features, such as de novo network enrichment (39) and to cover additional model organisms for which high-quality PPI networks are available.

## DATA AVAILABILITY

DIGGER is accessible at https://exbio.wzw.tum.de/digger. Source code is available at https://github.com/louadi/DIGGER. 3did interactions were downloaded from https://3did.irbbarcelona.org/ (version 2019_01). DOMINE interactions were downloaded from https://manticore.niehs.nih.gov/cgi-bin/Domine (version2.0). BioGRID interactions were downloaded from https://thebiogrid.org/ (version 3.5.187). The latest joint graph as well as the datasets used in DIGGER are available at https://exbio.wzw.tum.de/digger/download. The source code for the RNA-Seq analysis is available at (https://github.com/louadi/RNA-Seq-DIGGER).

## Supplementary Material

gkaa768_Supplemental_File
