# Supplementary material for: DIGGER: exploring the functional role of alternative splicing in protein interactions
Source: Nucleic Acids Res. 2020 Sep 25;49(D1):D309–18. doi: 10.1093/nar/gkaa768 (PMC7778957; doi:10.1093/nar/gkaa768)
Supplement: gkaa768_Supplemental_File [file gkaa768_supplemental_file.pdf]

Supplementary Information for

DIGGER: Exploring the functional role of alternative splicing in protein interactions

Zakaria Louadi[1], Kevin Yuan[1], Alexander Gress[2], Olga Tsoy[1], Olga Kalinina[2,3], Jan Baumbach[1,4], Tim Kacprowski[1,†,*], Markus List[1,†,*]

[1] Chair of Experimental Bioinformatics, Technical University of Munich, 85354 Freising, Germany
[2] Helmholtz Institute for Pharmaceutical Research Saarland (HIPS), Helmholtz Centre for Infection Research (HZI), 66123 Saarbrücken, Germany.
[3] Faculty of Medicine, Saarland University, 66421 Homburg, Germany
[4] Department of Mathematics and Computer Science, University of Southern Denmark, 5230 Odense M, Denmark

[†] joint last author
* To whom correspondence should be addressed. Tel: +49-8161-71-2761; Email: markus.list@wzw.tum.de
Correspondence may also be addressed to Tel: +49-8161-71-2710; Email: tim.kacprowski@wzw.tum.de

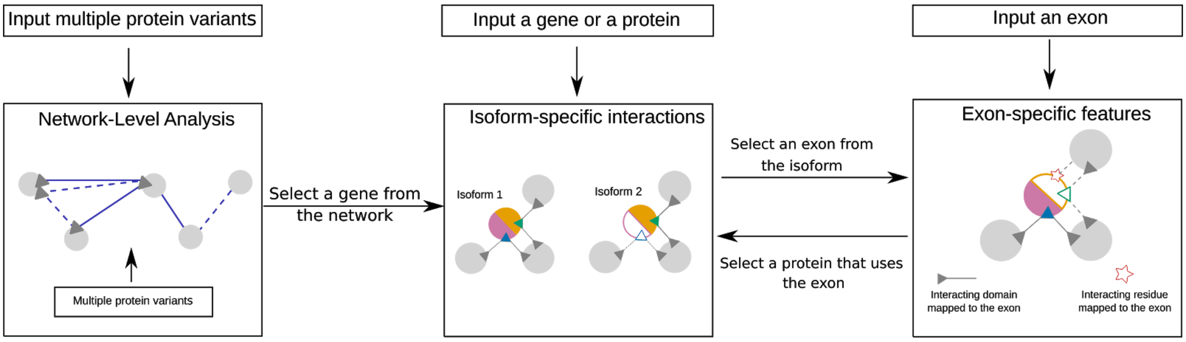

Supplementary Figure 1. Navigation through different modes of DIGGER.

Supplementary Table 1: Comparison of DIGGER to other tools. The main functionalities of these software tools are to use structural annotations of protein domains and their interactions to predict the effect of alternative splicing in the interactome.

**Features**	**DomainGraph** (1)	**DIIP** (2)	**PPIXpress** (3)	**DIGGER**
integrates PPIs with DDIs	✓	✓	✓	✓
graphical visualization	✓	-	-	✓
integrate interactions on a residue-specific level	-	-	-	✓
query isoform-specific interaction	-	✓ (2,944 proteins and 4,363 interactions)	-	✓ (9,370 proteins and 52,083 interactions)
query specific protein domain Interactions	✓	-	-	✓
query interactions between multiple protein variants	-	-	✓ output a network file	✓ visualization + network file
subnetwork construction	-	-	✓ whole proteome	✓ limited to visualize up to 2000 proteins
exon-level analysis	(✓) With AltAnalyze output	-	-	✓ for any annotated exon (Ensembl id or position in hg38)
consequence of exon skipping on PPI	(✓) assessed via visualization	-	-	✓ scored as a percentage of missing DDIs
result export	✓	-	✓	✓
supported species	12 species	human	4 species	human
dependencies / interface	Cytoscape + AltAnalyze	web tool	Java tool with GUI	web tool

Supplementary Table 2. Isoform-specific interactions reported by Yang *et al.* (4). The list contains isoforms resulting from splicing events that affect protein domains. The interacting domains identified by DIGGER are highlighted in green.

Entrez ID	Gene Symbol	Interacting isoform	Non-Interacting Isoform	Interactor Entrez ID	Interactor symbol	Interacting domains	Status
573	BAG1	BAG1_1	BAG1_2	3312	HSPA8	single	identified
598	BCL2L1	BCL2L1_1	BCL2L1_2	572	BAD	single	identified
598	BCL2L1	BCL2L1_1	BCL2L1_3	572	BAD	single	identified
835	CASP2	CASP2_1	CASP2_2	835	CASP2	single	identified
1020	CDK5	CDK5_1	CDK5_2	894	CCND2	single	Not identified
1876	E2F6	E2F6_1	E2F6_3	7027	TFDP1	multiple	identified
1876	E2F6	E2F6_1	E2F6_3	7029	TFDP2	multiple	identified
3932	LCK	LCK_1	LCK_3	5747	PTK2	multiple	identified
4690	NCK1	NCK1_2	NCK1_1	10152	ABI2	multiple	identified
4690	NCK1	NCK1_2	NCK1_1	10174	SORBS3	multiple	identified
4690	NCK1	NCK1_2	NCK1_3	10174	SORBS3	multiple	identified
4747	NEFL	NEFL_2	NEFL_1	1674	DES	single	identified
6257	RXRB	RXRB_1	RXRB_3	9971	NR1H4	multiple	identified
6257	RXRB	RXRB_1	RXRB_3	5915	RARB	multiple	identified
6257	RXRB	RXRB_1	RXRB_4	5914	RARA	multiple	identified
6257	RXRB	RXRB_1	RXRB_4	10062	NR1H3	multiple	identified
6257	RXRB	RXRB_1	RXRB_4	9971	NR1H4	multiple	identified
6257	RXRB	RXRB_1	RXRB_4	5915	NR1H4	multiple	identified
6257	RXRB	RXRB_1	RXRB_4	5915	RARB	multiple	identified
6257	RXRB	RXRB_2	RXRB_3	11043	MID2	single	identified
6257	RXRB	RXRB_2	RXRB_3	84993	UBL7	multiple	identified
6257	RXRB	RXRB_2	RXRB_3	9971	NR1H4	multiple	identified
6257	RXRB	RXRB_2	RXRB_3	5915	RARB	multiple	identified
6257	RXRB	RXRB_2	RXRB_4	11043	MID2	multiple	identified
6257	RXRB	RXRB_2	RXRB_4	84993	UBL7	multiple	identified
6257	RXRB	RXRB_2	RXRB_4	5914	RARA	multiple	identified
6257	RXRB	RXRB_2	RXRB_4	10062	NR1H3	multiple	identified
6257	RXRB	RXRB_2	RXRB_4	9971	NR1H4	multiple	identified
6257	RXRB	RXRB_2	RXRB_4	5915	RARB	multiple	identified
8440	NCK2	NCK2_1	NCK2_2	868	CBLB	multiple	identified
8440	NCK2	NCK2_1	NCK2_2	3937	LCP2	multiple	identified
8440	NCK2	NCK2_1	NCK2_2	10152	ABI2	multiple	identified
10181	RBM5	RBM5_2	RBM5_1	4222	MEOX1	single	identified
10494	STK25	STK25_1	STK25_2	5987	TRIM27	-	Not identified

10494	STK25	STK25_1	STK25_3	5987	TRIM27	-	Not identified
10772	SRSF10	SRSF10_2	SRSF10_1	4354	MPP1	-	Not identified
23552	CDK20	CDK20_3	CDK20_1	23321	TRIM2	-	Not identified
23552	CDK20	CDK20_3	CDK20_4	23321	TRIM2	-	Not identified
23552	CDK20	CDK20_3	CDK20_5	23321	TRIM2	-	Not identified
51135	IRAK4	IRAK4_1	IRAK4_2	4615	MYD88	-	Not identified
55324	ABCF3	ABCF3_1	ABCF3_2	5987	TRIM27	-	Not identified
55324	ABCF3	ABCF3_2	ABCF3_1	11030	RBPMS	-	Not identified
83549	UCK1	UCK1_5	UCK1_3	83549	UCK1	single	identified
151531	UPP2	UPP2_1	UPP2_3	151531	UPP2	single	identified
151531	UPP2	UPP2_1	UPP2_4	151531	UPP2	single	identified
151531	UPP2	UPP2_2	UPP2_4	151531	UPP2	single	identified

REFERENCES

1. Emig,D., Salomonis,N., Baumbach,J., Lengauer,T., Conklin,B.R. and Albrecht,M. (2010) AltAnalyze and DomainGraph: analyzing and visualizing exon expression data. *Nucleic Acids Res.*, **38**, W755–62.

2. Ghadie,M.A., Lambourne,L., Vidal,M. and Xia,Y. (2017) Domain-based prediction of the human isoform interactome provides insights into the functional impact of alternative splicing. *PLoS Comput. Biol.*, **13**, e1005717.

3. Will,T. and Helms,V. (2016) PPIXpress: construction of condition-specific protein interaction networks based on transcript expression. *Bioinformatics*, **32**, 571–578.

4. Yang,X., Coulombe-Huntington,J., Kang,S., Sheynkman,G.M., Hao,T., Richardson,A., Sun,S., Yang,F., Shen,Y.A., Murray,R.R., *et al.* (2016) Widespread Expansion of Protein Interaction Capabilities by Alternative Splicing. *Cell*, **164**, 805–817.
